# The influence of 17q21.31 and *APOE* genetic ancestry on neurodegenerative disease risk

**DOI:** 10.3389/fnagi.2022.1021918

**Published:** 2022-10-20

**Authors:** Nadia V. Harerimana, Alison M. Goate, Kathryn R. Bowles

**Affiliations:** ^1^Department of Genetics and Genomic Sciences, Icahn School of Medicine at Mount Sinai, New York, NY, United States; ^2^Ronald M. Loeb Center for Alzheimer’s Disease, Icahn School of Medicine at Mount Sinai, New York, NY, United States; ^3^Icahn Genomics Institute, Icahn School of Medicine at Mount Sinai, New York, NY, United States; ^4^Estelle and Daniel Maggin Department of Neurology, Icahn School of Medicine at Mount Sinai, New York, NY, United States

**Keywords:** *APOE*, 17q21.31, *MAPT*, haplotype, genetic ancestry, evolutionary genetics, non-European populations, neurodegeneration

## Abstract

Advances in genomic research over the last two decades have greatly enhanced our knowledge concerning the genetic landscape and pathophysiological processes involved in multiple neurodegenerative diseases. However, current insights arise almost exclusively from studies on individuals of European ancestry. Despite this, studies have revealed that genetic variation differentially impacts risk for, and clinical presentation of neurodegenerative disease in non-European populations, conveying the importance of ancestry in predicting disease risk and understanding the biological mechanisms contributing to neurodegeneration. We review the genetic influence of two important disease-associated loci, 17q21.31 (the “*MAPT* locus”) and *APOE*, to neurodegenerative disease risk in non-European populations, touching on global population differences and evolutionary genetics by ancestry that may underlie some of these differences. We conclude there is a need to increase representation of non-European ancestry individuals in genome-wide association studies (GWAS) and biomarker analyses in order to help resolve existing disparities in understanding risk for, diagnosis of, and treatment for neurodegenerative diseases in diverse populations.

## Introduction

The global prevalence of neurodegenerative diseases is likely to rise with the increasing life expectancy worldwide. Approximately 50 million people are currently affected by dementia (Nichols et al., 2019), which is estimated to increase to 130 million by 2050.^1^ Dementia is a major cause of disability, institutionalization, and mortality as well as a huge social and economic burden associated with the care of affected individuals (see text footnote 1). Among neurodegenerative diseases, Alzheimer’s disease (AD) is the most common and accounts for 60–70% of all cases (Seshadri and Wolf, 2007), whereas Parkinson’s disease (PD)/Lewy body dementia (LBD) is the second-most common neurodegenerative disorder, with a global prevalence of over 6 million (Nichols et al., 2019).

**FIGURE 1:**
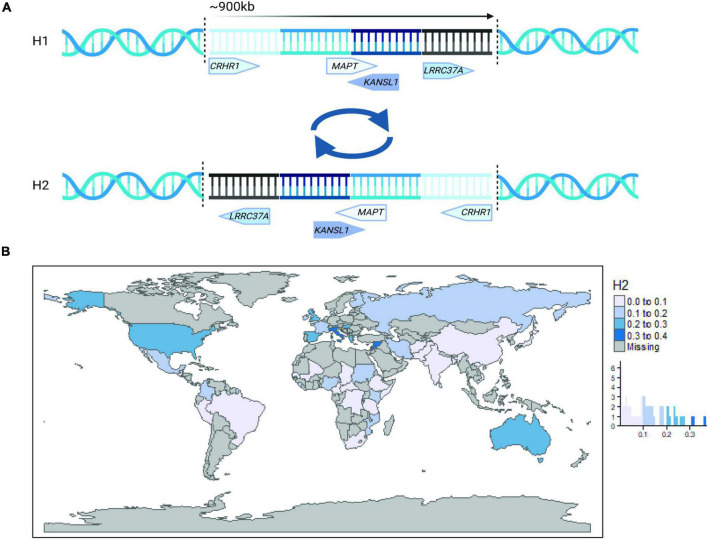
The 17q21.31 locus. **(A)** A simplified schematic illustration of the 17q21.31 inversion locus. The inversion breakpoints are represented by vertical dotted lines, and the approximate location and direction of four representative genes are presented for haplotype H1 (top) and H2 (bottom). **(B)** Worldwide 17q21.31 H2 allele frequency plotted by country and histogram. Haplotype frequency estimates were obtained from Donnelly et al. (2010), Steinberg et al. (2012), and publicly available 1000 Genomes Project data (www.internationalgenome.org).

During the previous few decades, large-scale genome-wide association studies (GWAS) have succeeded in uncovering the genetic landscape and pathophysiological processes involved in neurodegenerative diseases. In particular, we have learned that apolipoprotein E (*APOE*) is the major susceptibility gene for late-onset AD (Lambert et al., 2013) and LBD (Chia et al., 2021). Similarly, the 17q21.31 locus, which is a 970 kb region of high linkage disequilibrium (LD) encoding two distinct haplotypes (H1 and H2) and encompassing the *MAPT* gene (Figure 1A), has been genetically associated with several primary tauopathies, as well as PD (Höglinger et al., 2011; Kouri et al., 2015; Jun et al., 2016; Bandres-Ciga et al., 2019; Nalls et al., 2019). Although this accumulated knowledge has greatly expanded our understanding of neurodegenerative diseases, these studies have focused on populations of European ancestry, thus it remains unclear how this knowledge extends to and is applicable to estimation of disease risk and understanding of pathogenic disease mechanisms in other global populations.

To date, a large body of research has shown that genetic studies have not captured the level of diversity that exists globally, and neurodegenerative diseases are not an exception (Sirugo et al., 2019). Indeed, many European-based GWAS findings have not been replicated in other ancestral populations, thus making their findings less valuable and applicable across different populations. In this review, we discuss the evolutionary selection and differences in the genetic architecture of two important loci in neurodegenerative diseases, 17q21.31 and *APOE*, among diverse global populations. We also discuss the genetic associations of these loci with neurodegenerative diseases in non-European populations. Additionally, we discuss examples that illustrate why the inclusion of ethnically diverse populations in neurodegenerative genomic and biomarker studies will facilitate our understanding of the contribution of 17q21.31 and *APOE* to disease risk worldwide.

## Global population frequency and evolutionary selection

### 17q21.31 locus

The 17q21.31 H2 haplotype occurs at strikingly different frequencies across global populations (Figure 1B), with the highest frequencies occurring in Southern Europe and Southwest Asia (Donnelly et al., 2010). In contrast, it occurs at variable frequencies across Africa and is practically absent in East Asian populations (Stefansson et al., 2005; Donnelly et al., 2010; Alves et al., 2015). The source of this apparent positive selection in Europeans has been under debate since the identification of the structural inversion in 2005 (Cruts et al., 2005; Stefansson et al., 2005).

The inversion at 17q21.31 is most likely a result of non-allelic homologous recombination, facilitated by the presence of repetitive, low copy number repeats at the distal ends of the locus (Stefansson et al., 2005; Zody et al., 2008; Itsara et al., 2012; Dennis and Eichler, 2016). By comparing sequence similarities across haplotypes, global populations, and non-human primates, several estimates place the origin of the inversion event at ∼3 million years ago (Cruts et al., 2005; Stefansson et al., 2005), pre-dating modern *Homo sapiens*. Indeed, the H2 inversion has been identified in non-human primates and is highly polymorphic, indicative of multiple, repetitive inversion events over the last 10 million years of evolution that have increased in their copy number complexity in humans (Zody et al., 2008; Watson et al., 2014). This hypothesis is also supported by a 30 kbp region of striking similarity between H1 and H2 haplotypes surrounding the *CRHR1* gene at the 5′ end of the inversion, which is indicative of a possible double recombination event (Steinberg et al., 2012). However, another most common recent ancestor analysis based on haplotype structure rather than sequence similarity estimates a more recent inversion event occurring 16,400–108,400 years ago (Donnelly et al., 2010), which the authors argue correlates better with current global distributions of the H2 haplotype.

Interestingly, despite its low frequency, the inverted H2 allele has been proposed as the ancestral allele of African origin (Boettger et al., 2012; Steinberg et al., 2012), as evidenced by the presence of an H2 sub-haplotype lacking multiple duplication events (H2’) enriched in African hunter-gatherer populations compared to West African or European populations (Steinberg et al., 2012). Additionally, the H2 haplotype is much more homogeneous than H1, but greater diversity of H2 has been identified across African populations compared to Europeans (Stefansson et al., 2005; Alves et al., 2015). Given that the H2 inversion may have originated in Africa, it is curious why its frequency should be so low. The frequency of H2, combined with the striking homogeneity of H2 haplotypes in non-African populations, has led to the suggestion of a recent bottleneck and/or selective sweep following migration and gene flow out of Africa (Stefansson et al., 2005; Steinberg et al., 2012). However, more recent analyses suggest that there is little evidence for positive selection of H2 (Alves et al., 2015; Shimada and Nishida, 2020), and population differences may be explained by restricted recombination between haplotypes and demographic history, without the need for additional selective pressure (Steinberg et al., 2012; Alves et al., 2015).

Donnelly et al. (2010) highlighted that the high frequency of H2 in the Mediterranean raises the possibility of a Southwest Asian origin for the inversion, which would explain the comparatively high frequency of H2 in North African populations by gene flow back into Africa and would suggest that H2 haplotypes that originated in Africa could be in a non-inverted orientation. The inversion has been found to be highly polymorphic in Old World monkey species and Orangutan (Zody et al., 2008), and assessment of chromosome structure in H2 homozygote humans indicated heterozygosity of the inversion was present (Rao et al., 2010). However, this finding has not yet been replicated, possibly because studies confirming the H2 inversion status by fluorescent *in situ* hybridization or BAC cloning have utilized small samples from restricted ancestral populations (Stefansson et al., 2005; Gijselinck et al., 2006; Zody et al., 2008; Donnelly et al., 2010).

As well as variable H2 inversion frequencies across global populations, there are also highly variable frequencies of complex duplication events and copy number variants (CNVs) at the inversion breakpoints across populations on both haplotypes, but particularly on H1 (Boettger et al., 2012; Steinberg et al., 2012). Duplication events and CNVs are a major source of human genetic diversity by facilitating the creation of novel genes and regulatory elements (McElroy et al., 2009; Dennis and Eichler, 2016). It is thought that this is achieved by removing the ancestral selection restraint on a locus through the creation of genetic redundancy (Dennis and Eichler, 2016). It is therefore likely that variable CNVs on different H1 or H2 sub-haplotypes are likely to have altered functional effects, and that these are variable across different global populations. In 2012, two groups independently identified specific duplication events on the H1 haplotype that vary across populations (Boettger et al., 2012; Steinberg et al., 2012). Of note was the partial duplication of *KANSL1* that is much more frequent in Europeans compared to Africans or East Asians and results in the production of novel transcripts of unknown functional effect (Boettger et al., 2012). In contrast, increased copy number of a region encompassing *NSF*, which is upstream of *KANSL1*, appears to be more frequent in East Asian populations compared to Europeans and Africans (Steinberg et al., 2012).

Surprisingly, since the identification of complex CNVs at the 17q21.31 locus, very little has been done to understand the functional effects of these variants and their relevance to disease risk. Similarly, while the distribution of H1 and H2 haplotypes and sub-haplotypes vary across global populations, the contribution of this locus to neurodegenerative disease risk in non-European populations remains largely unexplored. However, given the structural complexity of the locus and complex evolutionary history that results in distinct population-specific structural, and presumably functional, differences, it is important to expand our investigation of the 17q21.31 locus beyond European ancestry to truly understand its function and contribution to disease risk.

### APOE

**FIGURE 2:**
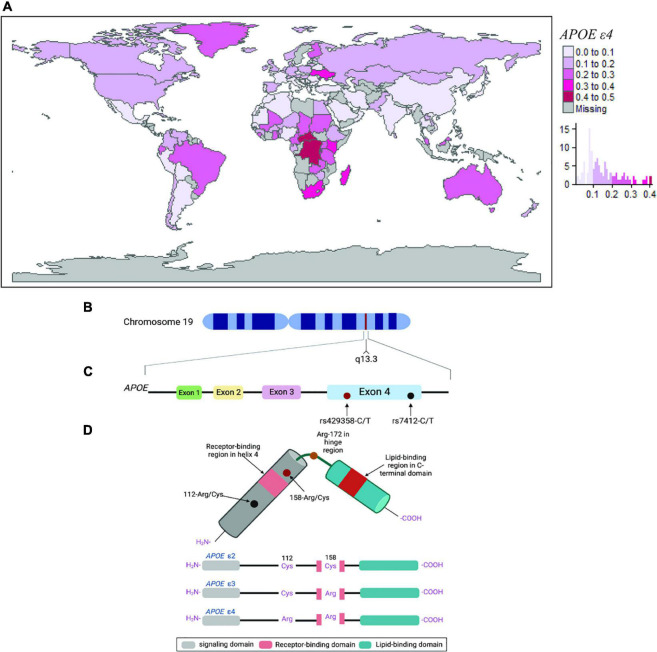
The *APOE* ε4 locus. **(A)** Worldwide *APOE* ε4 allele frequency plotted by country and by histogram. Allele frequency estimates were obtained from Singh et al. (2006) and other literature search (see Supplementary Information). **(B–D)** Schematic representation of the *APOE* ε4 locus. **(B)** The structure and location of the *APOE* gene on chromosome 19. **(C)** Estimated structure of the *APOE* protein. **(D)** The three main *APOE* isoforms ε2, ε3, and ε4, respectively, are the result of non-synonymous polymorphisms that cause amino acid changes at positions 112 and 158 of the *APOE* protein.

Similar to the 17q21.31 H2 haplotype, the distribution of different *APOE* alleles varies considerably across the world (Figure 2A). The ε3 allele is the most common isoform globally, with the highest allele frequency in Asia, Europe, and Africa in descending order (Singh et al., 2006). *APOE* ε2 is the least common isoform and is markedly higher in frequency in South African Zulu (19%) and Eurasian (18%) populations (Chikosi et al., 2000; Singh et al., 2006). Unlike in European populations and in contrast to the 17q21.31 H2 haplotype, the *APOE* ε4 allele is relatively highly abundant in central and southern regions of Africa, mainly in populations such as Pygmies (41%) and Khoisan (37%) (Corbo and Scacchi, 1999).

The *APOE* gene is widely expressed in all vertebrates (Duggan and Callard, 2001), but the ε4 allele has been only observed in chimpanzees and humans (Hanlon and Rubinsztein, 1995; Vamathevan et al., 2008). In early humans, it is thought that the origin of the ε4 allele correlates with the increased requirements for endurance in locomotion (i.e., physical exercise) around 1.8 million years ago during the development of a hunter-gatherer lifestyle, whereas the ε2 and ε3 alleles are estimated to date back to only 200,000–300,000 years ago (Raichlen and Alexander, 2014). *APOE* isoforms are thought to have originated in a South Asian subpopulation, followed by the westward migration of modern humans to Asia, Africa, Europe, and then North and South America. This hypothesis is supported by the relatively high frequency of ε3 in Asia (85%) and the near absence of ε2 in Amerindian populations from North and South America (4.6–4.9%) (Singh et al., 2006). The historical allelic divergence, combined with the relatively high frequency of the ε4 allele in populations where an economy of hunting still exists, or food supply has been sporadically available, indicates that ε4 is likely to be the ancestral allele (Hanlon and Rubinsztein, 1995; Seixas et al., 1999).

The human *APOE* gene is located on chromosome 19 at position q13.3 (Figures 2B,C) and encodes for a 299 amino acid protein (∼34 kDa) with multiple functions, particularly with cholesterol metabolism, lipid homeostasis, and innate immunity (Davignon et al., 1988), which are all likely to contribute to increased reproductive success and protection against infection load. Furthermore, *APOE* isoforms differ by a unique amino acid combination at positions 112 and 158: ε2 (Cys112, Cys158), ε3 (Cys112, Arg158), and ε4 (Arg112, Arg158), which modify their structure and function (Figure 2D). In regard to lipoprotein-binding preferences, *APOE* ε4 has a high affinity to very low-density lipoprotein (VLDL) particles (Mahley and Rall, 2000) while the ε2 has poor binding affinity to low-density lipoprotein (LDL), which are both associated with increased plasma cholesterol and triglycerides (Zhao et al., 2018). In addition, the ε4 allele has an exposed Arg-61 that is known to interact with Glu-255, which does not occur in the other isoforms, and has been suggested to represent another underlying factor to the adverse effects of the ε4 allele (Raffaï et al., 2001). Interestingly, mouse *APOE* ε4 lacks the equivalent of Arg-61 and resembles the ε3 allele in terms of lipoprotein-binding preferences (Raffaï et al., 2001).

Several hypotheses have been formulated about the functional and potential selective pressures contributing to the successful adaptation of the ε4 allele, including climate conditions, conservation practices, and infection load (Corbo and Scacchi, 1999). Consistent with the notion that meat eating (i.e., increased dietary fats) improves adaptive responses to pathogens and facilitates reproduction in populations where infections are highly prevalent, it is noted that fertility and fecundity were found to be higher in *APOE* ε4 allele carriers in the indigenous populations of South America (Corbo et al., 2004), West Africa (van Exel et al., 2017), and rural individuals of Western Europe (Jasienska et al., 2015). This hypothesis is also supported by the predominant presence of ε4 in indigenous people of Central Africa (40%), Australia (26%), Oceania (49%), and South America (27%) (Corbo and Scacchi, 1999; Singh et al., 2006).

In contrast, positive selection for the *APOE* ε4 allele in European populations is thought to reflect adaptations to extreme climate conditions (Eisenberg et al., 2010; Huebbe and Rimbach, 2017), such as high-latitude cold environments [e.g., Northern latitudes; (Lovegrove, 2003; Froehle and Schoeninger, 2006)] or low-latitude hot environments [e.g., near the equatorial level; (Eisenberg et al., 2010; Huebbe et al., 2011)], where human energy expenditure is known to be higher due to an increased requirement for thermoregulation. In Europe and Asia, ε4 allele distribution appears to follow a North-to-South latitudinal gradient, with a fourfold higher frequency in the North (e.g., >25% in Finland) than in the Mediterranean or South Asia area (e.g., <10% in Sardinia) (Egert et al., 2012). The most likely explanation is that high temperatures may influence demand for cholesterol indirectly via metabolism increase, thus promoting the accumulation of the ε4 allele. In this context, the *APOE* ε4 allele is also thought to protect against vitamin D deficiency via better absorption of fat-soluble vitamin D in geographical regions, such as northern Europe, subjected to diminished sunlight exposure (Gerdes, 2003; Huebbe and Rimbach, 2017).

Similar to the 17q21.31 locus, although there has been investigation with regard to the evolution and global distribution of *APOE* alleles, there are sparse data and inconsistent findings on the role of the ε4 allele in relation to neurodegenerative disease risk across global populations. However, understanding the contribution of *APOE* to disease risk in non-European populations is essential to uncover the biological mechanisms underlying global disease in order to develop appropriate and effective therapeutics for different populations.

## Genetic contribution to neurodegenerative disease in non-European populations

### 17q21.31

The 17q21.31 locus has been genetically associated with 113 different traits across 176 studies (Buniello et al., 2019).^2^ Of these studies, only 12% included analysis of Asian, African, or Hispanic populations, and did not include assessment of any neurological, psychiatric, or neurodegenerative phenotypes. However, in European populations, the 17q21.31 locus has been genetically associated with several primary tauopathies, including progressive supranuclear palsy (Höglinger et al., 2011), corticobasal degeneration (CBD) (Kouri et al., 2015), frontotemporal dementia (FTD) (Ferrari et al., 2019), as well as the secondary tauopathy AD (Jun et al., 2016; Bellenguez et al., 2022) and Parkinson’s disease (Bandres-Ciga et al., 2019; Nalls et al., 2019), with the H2 haplotype consistently conferring protection against disease risk (Table 1). Given that Europeans account for an estimated 73% of all individuals included in genetic studies (Sirugo et al., 2019), it is therefore likely that the lack of association of the 17q21.31 locus with neurodegenerative disease in non-European populations is due to a paucity in data and analyses, especially for rarer diseases such as PSP.

**TABLE 1:** Summary of 17q21.31 genetic association with neurodegenerative disease across global populations.

Phenotype	Population/consortium	Ancestry	Approach	*N*	Top SNP/variant	Effect size (OR/beta)	*P-value*	References
*APOE* ε4-AD	IGAP	European	GWAS	70,721	rs2732703	0.73	5.8 × 10^– 9^*	Jun et al., 2016
AD	EADB, UK Biobank, ADGC, FinnGen, CHARGE	European	GWAS	788,989	rs199515	0.94	9.3 × 10^– 13^	Bellenguez et al., 2022
AD	Spain, Uruguay	South American	Targeted NGS	172		A152T, S318L		Jin et al., 2012
AD	MVP, ADGC	African, European	GWAS	80954		–		Sherva et al., 2022
AD	ADGC	African, European	GWAS	8,006		–		Kunkle et al., 2021
AD	China	East Asian	GWAS	11,506		–		Jia et al., 2021
AD	Japan	East Asian	GWAS	11,698		–		Shigemizu et al., 2021
FTD, AD	GIFT, USA	European	Targeted sequencing	15,369	A152T	3 (FTD), 2.3 (AD)	0.0005 (FTD), 0.004 (AD)	Coppola et al., 2012
AD, FTD, PSP	GIFT	European	Exome array	664	rs8070723 (PSP)	0.1796	0.0056	Chen et al., 2015
PSP	USA	European	GWAS	9,706	rs8070723	5.46	1.5 × 10^– 116^*	Höglinger et al., 2011
CBD	USA	European	GWAS	3,987	rs393152	3.7	1.42 × 10^– 12^*	Kouri et al., 2015
FTD	Brazil	South American	Targeted sequencing	76	N279K, IVS10 + 16			Takada et al., 2016
PD	Spain	European	GWAS	7,849	rs113434679	−0.311T	8.57 × 10^– 13^	Bandres-Ciga et al., 2019
PD	IPDGC, PDWBS, SGPD, UK Biobank, 23andMe	European	GWAS	1,456,300	rs62053943	−0.27T	3.58 × 10^– 68^	Nalls et al., 2019
PD	LARGE-PD	South American	GWAS	1,497	rs1800547	−0.432T	0.001	Loesch et al., 2021
PD	South African	European, African	Targeted sequencing	202	IVS3 + 18, A90A, IVS4 + 9, A562A, N590N, P605P, IVS11 + 40			Keyser et al., 2011
PD	South African, Nigerian	African	Targeted NGS	47	G213A, A285A, S318L, T441H, A495T, S533T + insertion, P606P			Oluwole et al., 2020
PD	Singapore, Hong Kong, Taiwan, China, South Korea, Japanese	East Asian	GWAS	31,575		–		Foo et al., 2020
PD	Chinese Han	East Asian	Targeted genotyping	962		–		Fan et al., 2021

^T^Effect size reported as Beta.

*Statistic derived from joint/meta-analysis of multi-stage study.

^●^Indicates large meta-analysis study.

Dashes indicate no association with the 17q21.31 locus was identified or reported. IGAP, international genomics of Alzheimer’s project; EADB, European Alzheimer and Dementia Biobank; ADGC, Alzheimer’s disease genetics consortium; CHARGE, cohorts for heart and aging research in genomic epidemiology; MVP, million veterans program; GIFT, genetic investigation in frontotemporal dementia; IPDGC, international Parkinson’s disease genetics consortium; PDWBS, Parkinson’s disease web based study; SGPD, systems genomics of Parkinson’s disease consortium; LARGE-PD, Latin American Research Consortium on the Genetics of Parkinson’s disease; NGS, next-generation sequencing.

However, given global population differences in 17q21.31 structure, variation, and haplotype frequency, it is also possible that the lack of replication of genome-wide association signals at this locus for more common disorders, such as AD and PD, is a result of varied genetic architecture in non-European populations. For example, two recent PD GWAS conducted in Chinese Han and Japanese populations do not replicate the 17q21.31 association consistently observed in Europeans (Foo et al., 2020; Fan et al., 2021). This is not surprising, as the protective H2 haplotype is absent in these populations, and the relatively small sample sizes compared to European-ancestry consortia studies may preclude the identification of less common H1 sub-haplotypes that contribute to disease risk. To our knowledge, there are currently no PD GWAS that have been conducted in African ancestry populations; however the IPDGC-Africa consortium^3^ is addressing this gap in our understanding of PD genetics. Interestingly, African ancestry in South American Latino populations has been found to be protective against PD risk (Loesch et al., 2021), although this was not specific to the 17q21.31 locus. The same study also replicated the European effect size and direction of the 17q21.31 association with PD risk in South Americans, although due to a small sample size, the association did not reach significance (Loesch et al., 2021). Regardless, this is an indication of the relevance of the 17q21.31 locus to PD risk in non-European and admixed populations that warrants further investigation.

Despite the lack of PD GWAS, several groups have attempted to identify novel *MAPT* variants that may contribute to PD risk in African populations. For example, Keyser et al. (2011) identified two novel *MAPT* variants in South African individuals with PD (A91V and V635I), although these were predicted to be benign (Keyser et al., 2011). Similarly, Oluwole et al. (2020) identified an additional seven novel *MAPT* variants in black South African and Nigerian individuals that may be associated with PD risk, with unknown functional effects (Oluwole et al., 2020). While these data indicate the presence of ancestry-specific variants of *MAPT*, the association with PD risk is less convincing; this is unsurprising given that the GWAS association in European populations spans a ∼1 Mb region, which incorporates numerous genes other than *MAPT*. There is also limited evidence that the 17q21.31 signal implicates *MAPT* as the causative gene, but rather recent studies favor *KANSL1*, *CRHR1*, or *LRRC37A2* as likely candidates contributing to PD risk (Loesch et al., 2021; Yao et al., 2021; Bowles et al., 2022). The contribution of rare variants in these specific genes has not yet been investigated in any population.

While Alzheimer’s disease is neuropathologically defined by the presence of neurofibrillary tau tangles, the genetic association of the 17q21.31 locus with AD risk is less clear. Until recently, an association signal within either *MAPT* or across the 17q21.31 locus has been notably absent, with the exception of in *APOE* ε4- populations specifically (Jun et al., 2016). However, it has just reached genome-wide significance in the most recent and largest European AD GWAS, independent of *APOE* genotype (Bellenguez et al., 2022). In addition, the rare *MAPT* A152T variant has been associated with the development of an AD-like dementia (Coppola et al., 2012), suggesting that the locus may play a role in modifying AD risk. Consistent with this, the *MAPT* A152T and S318L mutations were observed to occur 3× as frequently in AD cases compared to controls in an Ibero-American cohort, with Spanish and Uruguayan ancestry (Jin et al., 2012), with additional missense variants identified in exons 4a, 7, and 10 with unknown effects (Jin et al., 2012).

Several large GWAS have been conducted in non-European ancestry populations for AD, including the largest African-American AD GWAS to date, which utilized data derived from the Million Veterans Program (Sherva et al., 2022), as well as a recent publication from the ADGC (Kunkle et al., 2021). Large-scale studies have also been conducted in East Asian populations from China and Japan (Jia et al., 2021; Shigemizu et al., 2021). Similar to previous, smaller European ancestry studies, the 17q21.31 locus was not associated with AD risk in any of these GWAS. However, assessment of the 17q21.31 association in *APOE* ε4- individuals specifically may provide additional power in studies of non-European populations as they have done in Europeans (Jun et al., 2016), but this has been overlooked. Similarly, assessment of the 17q21.31 locus in fine-mapping and local ancestry analyses of known AD loci in non-European populations has been excluded for the same reason. As such, *APOE* genotype stratification analyses in these groups remain to be carried out in order to determine the contribution of the 17q21.31 locus and *MAPT* to AD risk.

Primary tauopathies, such as PSP and FTD, have been more challenging to investigate by GWAS due to their relative rarity compared to AD and PD. However, the effect size of the 17q21.31 locus is much larger for these disorders (e.g., odds ratio ∼4 compared to ∼1.5 for PD) (Höglinger et al., 2011; Nalls et al., 2019), thus necessitating smaller studies to observe a significant association in this region. Furthermore, rare autosomal dominant mutations in *MAPT* have demonstrated the direct relevance of this gene and locus to FTD/PSP pathogenesis.^4^ Given the relatively low frequency of PSP and FTD, little has been done to investigate the contribution of the locus in non-European populations. For example, Chen et al. (2015) embarked on a multi-ancestral exome array study of AD, FTD, and PSP, but there were not sufficient FTD or PSP cases available to carry out the multi-ancestry replication analyses (Chen et al., 2015).

Frontotemporal dementia has been reported as being less common in Asia than in Europe, and fewer individuals with FTD reported a positive family history of the disorder (9.5–20%) compared to Europeans (30–50%) (Ng, 2015), indicating that there may be a reduced genetic contribution of the 17q21.31 locus to disease risk in Asia. However, as epidemiological and clinical studies have also suggested there could be a slightly higher prevalence of PSP in Japan compared to European populations (Takigawa et al., 2016), the prevalence of primary tauopathies in East Asia requires additional investigation. In either case, given the absence of the protective H2 haplotype in these populations, understanding the genetic contribution of the 17q21.31 locus to FTD and PSP in Asia will likely provide valuable insight into the pathogenic mechanisms specific to H1 that underlie these diseases. Additionally, a common sub-type of PSP has been described that is defined by the presence of cerebellar ataxia, which appears to be specific to Asia (Krzosek et al., 2022), and suggests that there is likely to be ancestry-specific variation across the 17q21.31 locus H1 haplotype in East Asian populations that may differentially contribute to disease risk and clinical phenotypes.

Numerous rare *MAPT* variants associated with FTD have been identified in China and Japan (Kasuga et al., 2015; Ng, 2015). *MAPT* has been reported as the most common pathogenic FTD gene in China (Jiang et al., 2021), compared to the *C9ORF72* expansion that is most prevalent in European familial FTD cases (Smith et al., 2013). Numerous autosomal dominant FTD *MAPT* mutations have also been identified in Colombia at a relatively high frequency compared to other populations (Takada et al., 2016; Zuluaga-Castaño et al., 2021; Acosta-Uribe et al., 2022), which is likely due to multiple founder events and population bottlenecks arising from Spanish invasion and African enslavement, followed by outbreaks of infectious disease (Acosta-Uribe et al., 2022). However, the relative abundance of *MAPT* variants identified in Colombia may also reflect the substantial efforts being conducted by large initiatives focused on identifying AD and FTD mutations in this population specifically. Regardless, given the frequency of pathogenic rare variants in this population, it would be of interest to assess the impact of this history and admixture on common 17q21.31 variants and structure and resulting function and contribution to disease risk that may be unique to this group.

### APOE

The *APOE* locus is a well-established genetic risk factor for Alzheimer’s disease (AD) (Corder et al., 1993). Given the evolutionary shifts of this gene, the *APOE* alleles present a pronounced stepwise effect (ε2 < ε3 < ε4), where the ε2 allele confers substantial protection for AD and the ε4 allele increases risk in European ancestry populations [ε4/ε4 odds ratio (OR):12.5], but not conclusively in other ethnic groups (Farrer et al., 1997; Tang et al., 1998; Reiman et al., 2020; Table 2). In African populations, the genetic association of the ε4 allele with AD risk is weak or sometimes absent, but relatively present in African American individuals (ε4/ε4 OR: 5.7) (Farrer et al., 1997). The most recent and largest genome-wide association meta-analysis of African American individuals also confirmed *APOE* as the strongest risk factor of AD in this group (Sherva et al., 2022). This may be attributable to the presence of European admixture in African Americans, thus accounting for the population differences in the effect of the ε4 allele. Furthermore, African populations harbor the greatest diversity in the global population, yet only <2% of GWAS have included the African genome (Sirugo et al., 2019); thus, there may be additional protective variants that alleviate the effect of the ε4 allele in African populations yet to be identified. Additionally, the differential effect of the *APOE* ε4 allele in non-European admixed populations appears to be explained by the local ancestral background on which the allele lies; *APOE* ε4 alleles of African ancestry confer lower risk than those of European ancestry (Rajabli et al., 2018). In other words, individuals who have the *APOE* ε4 allele derived from an African ancestor have a lower AD risk as observed in the African population, while those who have inherited their *APOE* ε4 alleles from a European ancestor have the AD risk observed in European populations.

**TABLE 2:** Summary of *APOE* locus genetic association with neurodegenerative disease across global populations.

Phenotype	Population/consortium	Ancestry	Approach	*N*	Top SNP/variant	Effect size (OR/beta)	*P-value*	References
AD	Chinese	East Asian	GWAS	70,721	rs73052335	0.870	1.44E-14	Zhou et al., 2018
AD	Chinese	East Asian	GWAS	4,069	rs439401	1.5	2.06 × 10^– 6^	Jia et al., 2021
AD	South Korea	East Asian	GWAS	500	rs429358	4.81	2.59 × 10^– 33^	Park et al., 2021
AD	South Korea	East Asian	GWAS	2,291	rs429358	2.608	3.74 × 10^– 43^	Kang et al., 2021
AD	JGSCAD, South Korea, and ADGC	East Asian	GWAS	2,024	rs7519866	0.71	9.70 × 10^– 6^*	Miyashita et al., 2013
AD	Japan	East Asian	GWAS	8,808	rs769449	4.01	9.04 × 10^– 22^	Hirano et al., 2015
AD	WHICAP, EFIGA	Caribbean hispanic	Targeted Genotyping	1,084		–		Lee et al., 2011
AD	ADGC	African American	GWAS	5,896	rs429358	2.31	5.5 × 10^– 47^	Reitz et al., 2013
AD	Belgium, Finland, France, Italy, and Spain	European	GWAS	8,260	rs2965109	–	1,70E-09*	Lambert et al., 2009
AD	USA, UK, Germany, and Greece	European	GWAS	11,025	rs2075650	2.53	1.8 × 10^– 157^*	Harold et al., 2009
AD	ADC, Miami Brain Bank, USA, UK, and Netherlands Brain banks	European	GWAS	1,594		–		Corneveaux et al., 2010
AD	CHARGE, TGEN, EADI, GERAD	European	GWAS	14,283		–		Seshadri et al., 2010
AD	ADGC, ADC	European	GWAS	21,165	rs4420638	3.64	1.1 × 10^– 266^*	Naj et al., 2011
AD	ADGC, CHARGE, EADI, GERAD	European	GWAS	54,162		–		Lambert et al., 2013
AD	ADGC, CHARGE, EADI, GERAD	European	GWAS	63,926		–		Kunkle et al., 2019
AD	UKB	European	GWAS	368,440		–		Marioni et al., 2018
AD	PGC-ALZ, IGAP, ADSP, and UKB	European	GWAS	455,258		–		Jansen et al., 2019
AD	GR@ACE, IGAP, UKB, EADB, PGC-ALZ, ADGC, and GBCS	European	Meta-GWAS	409,435		–		de Rojas et al., 2021
AD	IGAP, EADB, UKB, ADGC, FinnGen, ANMerge, DemGene, TwinGene, STSA, Gr@ce, HUNT, BioVU, 23andMe, Gothenburg	European	GWAS	1,126,563		–		Wightman et al., 2021
AD	EADB, UKB, ADGC, FinnGen, CHARGE	European	GWAS	48,511		–		Bellenguez et al., 2022
AD	MVP, ADGC	African, European	GWAS	80,954		–		Sherva et al., 2022
AD	ADGC	African, European	GWAS	8,006		–		Kunkle et al., 2021
FTD	France, Greece, Ireland, Italy, Luxembourg, Spain, UK, Canada, USA	European	GWAS		rs429358	–	1.37 × 10^– 7^*	Mishra et al., 2017
LBD	USA, Canada, UK, Spain, France, Belgium, Netherlands, Denmark, Germany, Sweden, Italy, Australia	European	GWAS	6,638	rs769449	2.46	4.65 × 10^– 63^	Chia et al., 2021
AD	Japan	East Asian	GWAS	11,698		–		Shigemizu et al., 2021

*Statistic derived from joint/meta-analysis of multi-stage study.

^●^Dashes indicate no association with the APOE locus was not reported. JGSCAD, Japanese Genetic Study Consortium for Alzheimer’s disease; IGAP, International Genomics of Alzheimer’s project; EADB, European Alzheimer and Dementia Biobank; ADGC, Alzheimer’s disease genetics consortium; CHARGE, Cohorts for Heart and Aging Research in Genomic Epidemiology; MVP, Million Veterans Program; ADC, Alzheimer’s Disease Cooperative Study; WHICAP, Washington Heights-Inwood Columbia Aging Project; EFIGA, Estudio Familiar de Influencia Genetica en Alzheimer; TGEN, Translational Genomics Research Institute; EADI, European Association of Development Research and Training Institutes; GERAD, Genetic and Environmental Risk in Alzheimer’s Disease; UKB, Genetic and Environmental Risk in Alzheimer’s Disease; EADB, European DNA bank; GR@CE, Genome Research at Fundació ACE; HUNT, The Trøndelag Health Study.

In contrast to the variable effect of *APOE* ε4 observed in African ancestry populations, East-Asian population seems to be the most susceptible to the effect of the ε4 allele on AD risk, with a higher odds ratio compared to European and other non-European populations (ε4/ε4 OR:33.12) (Farrer et al., 1997). A series of large-scale AD GWAS in East Asian individuals from Japanese (Shigemizu et al., 2021), Chinese (Jia et al., 2021), and South Korean cohorts (Kang et al., 2021) have all confirmed the *APOE* locus as the most significant contributor to AD risk, notably with higher odds ratios compared to European ancestry individuals. It is worth noting that the larger effect size in East Asians may be due to the differences in allele frequency such that the proportional of *APOE* ε4 frequency differs between cases and controls, thus resulting in a larger odds ratio even though the total difference in the allele frequency is similar across populations (Choi et al., 2019). This means that, compared to the European population, the effect of the ε4 allele on AD risk is stronger in East Asians who have a lower ε4 frequency and weaker in African populations who have a higher ε4 frequency. Alternatively, Choi et al. (2019) also point out the TT genotype within the *APOE* promoter, the SNP rs405509, is highly frequent in East Asians and possibly accounts for the observed high magnitude of the effect of ε4 on AD risk (Choi et al., 2019). Nonetheless, it would be interesting to assess different East Asian cohorts individually, as these cohorts are often conducted as part of large meta-analyses, which often fail to detect small effects loci that could be unique in each country.

The genetic association of *APOE* with AD risk in Hispanic\Latino populations is less clear. An association of the *APOE* ε4 allele and AD is remarkably absent in even the largest AD GWAS performed to date in Hispanic\Latino populations (Lee et al., 2011), which included admixed individuals from the Dominican Republic and Puerto Rico, indicating that the locus may not be a main genetic contributor. However, significant associations between *APOE* ε4 and AD risk have been observed in candidate genetic studies, but with smaller effect sizes relative to studies of European ancestry populations (ε4/ε4 OR: 2) (Farrer et al., 1997). Indeed, Hispanics/Latinos are highly admixed, with >90% of their ancestry derived from native Americans, African slave, and European invasion. Thus, disentangling the relationship between *APOE* and AD continues to be challenging in part due to the heterogeneity of the Hispanic\Latino population. As such, an Ameridian ancestry on *APOE* ε4 locus has been found to confer protection in Colombians (Moreno-Grau et al., 2018) and Brazilians (Benedet et al., 2012), while contributing to increased risk of AD in Peruvians (Marca-Ysabel et al., 2021). Interestingly, African ancestry *APOE* is thought to be protective in a Caribbean Hispanic cohort (Blue et al., 2019). Given the complex relationships between *APOE* genotype, ancestry, and AD risk across Hispanic\Latino populations, it is worth pointing out that local ancestry, allele frequencies, and patterns of LD should be considered when predicting the genetic risk of AD in populations with non-European ancestry.

*APOE* ε4 has been found to increase the risk of dementia with Lewy bodies (DLB) (Chia et al., 2021) and FTD (Mishra et al., 2017). However, the overlap with AD clinical and neuropathological symptoms has made it challenging to recruit patients with pure DLB and/or FTD, respectively. Only a few European studies have attempted to do so. The most recent and largest GWAS of DLB detected the *APOE* locus as the top GWAS association (Chia et al., 2021). Similarly, a gene-based association study led by Mishra et al. (2017) using GWAS summary files from the international FTD consortium confirmed that the *APOE* ε4 allele increases the risk of behavioral variant of FTD (bvFTD). Given that DLB and FTD clinically and pathologically resemble AD and PD, this has hindered the collection of large cohorts of cases whose diagnosis is certain, and as a consequence, could also be restraining the presence of large-scale GWAS in non-European populations (Orme et al., 2018). Furthermore, it is important to note that the GWAS of DLB and FTD are not as large as those for AD, and the heterogeneity of FTD subtypes further complicates these studies and reduces sample size and power. Despite this link with DLB and FTD, *APOE* has not clearly been associated with other neurodegenerative diseases, implying that *APOE* is not a major contributor to disease risk for these disorders. However, these associations remain to be conducted in non-European populations to allow the discovery of population-specific genetic factors.

## 17q21.31 and *APOE* genetic ancestry on measurement of Alzheimer’s disease biomarkers

Biomarkers for Alzheimer’s disease, including cerebrospinal fluid (CSF) and plasma levels of Aβ40, Aβ42, total tau (t-tau), and phosphorylated tau (p-tau-181/217), as well as neuroimaging measures such as PET and MRI, are important for an accurate clinical diagnosis of dementia, determination of disease progression, and serve as key outcome measures for clinical trials of novel therapeutics. Importantly, many of these biomarkers have been found to vary both by genetic variation at the *APOE* and 17q21.31 loci, as well as by race, thus implying a likely influence of genetic ancestry on disease pathogenesis and subsequent interpretation of biomarker results.

*APOE* ε4 has been associated with increased levels of both CSF and circulating t-tau and p-tau-181, as well as reduced plasma Aβ42 in European ancestry populations (Mattsson et al., 2018; Morris et al., 2019; Brickman et al., 2021; Deniz et al., 2021). However the relationship between *APOE* ε4 and markers of AD in African ancestry populations appears to be less well defined. Deniz et al. (2021) found that the *APOE*ε4 allele was associated with reduced t-tau in plasma from African American participants, although consistent with European ancestry data, the levels increased with age and AD diagnosis (Deniz et al., 2021). Also consistent with European data, CSF and plasma Aβ42 appear to be reduced with *APOE*ε4 genotype in both African and Asian ancestry populations (Cruchaga et al., 2013; Nakamura et al., 2018; Morris et al., 2019; Brickman et al., 2021; Deniz et al., 2021). However, a recent study of an admixed Brazilian cohort demonstrated not only that an increased proportion of African ancestry was associated with reduced neuritic plaque burden but that *APOE* ε4 was only associated with worse cognition and more severe neuropathology when it was of European origin (Naslavsky et al., 2022). Furthermore, a study of amyloid PET positivity in cognitively normal individuals revealed that self-reported black participants had reduced signal compared to whites, and this effect was larger in *APOE* ε4 carriers (Deters et al., 2021).

A race × *APOE* interaction for CSF t-tau and p-tau-181 has also been reported; while these markers were lower in African American participants compared to Europeans overall, this difference was driven only by *APOE* ε4 carriers (Howell et al., 2017; Garrett et al., 2019; Morris et al., 2019; Choudhury et al., 2021). These findings appear to be consistent with the GWAS data described above, in that there appears to be a reduced contribution of *APOE* ε4 to AD risk and pathogenesis in African ancestry populations compared to Europeans. In contrast, a recent multi-ethnic community study reported higher levels of p-tau-181 and p-tau-217 in plasma associated with the *APOE* ε4 genotype in White, Hispanic, and Black individuals, but that the accuracy of p-tau in classifying AD diagnosis was improved in Hispanic and Black participants compared to white participants (Brickman et al., 2021). These inconsistencies may be due to the relatively small sample sizes used to assess biomarker abundance in non-European ancestry populations, which limits the generalizability and reproducibility of results. Regardless, it is apparent that genetic ancestry at the *APOE* locus is likely influencing the interaction with amyloid and tau accumulation and phosphorylation, which has implications for the use of these biomarkers in diagnostic criteria of AD in diverse populations.

The contribution of genetic variation at the 17q21.31 locus to AD biomarker detection has received less attention than *APOE* genotype; however, a recent GWAS meta-analysis revealed that the locus was significantly associated with plasma t-tau in European Americans but not in African Americans (Sarnowski et al., 2022), which is suggestive that genetic architecture related to ancestry may influence tau pathology. Histopathological studies have reported no effect of race on Braak score postmortem (Sandberg et al., 2001; Naslavsky et al., 2022), although a more recent study identified an interaction between ancestry, tau burden, and dementia severity (Naslavsky et al., 2022). It is possible that this lack of effect of ancestry on tau pathology is a result of underpowered studies or may require a more comprehensive analysis applying modern neuropathological techniques combined with local ancestry analyses across the 17q21.31 locus to investigate this fully. While a more recent PET study identified higher 18F-Flortaucipir binding, indicative of increased tau pathology, in the choroid plexus and hippocampus of African American individuals compared to European ancestry individuals, it was hypothesized that the signal was likely due to increased tracer off-binding to melanin in the choroid plexus, which was also likely to have influenced the hippocampal signal (Lee et al., 2018). Therefore, the relationship between CSF and plasma tau to pathology in the brain in African ancestry individuals is unclear.

Interestingly, 17q21.31 variants associated with either CSF or plasma t-tau have not been H1/H2-defining tag SNPs, but rather have tagged specific sub-haplotypes of the major H1 allele (Laws et al., 2007; Kauwe et al., 2008; Sarnowski et al., 2022), which confers risk for AD (Bellenguez et al., 2022). The H1c tag SNP, rs242557, showed the strongest association with plasma t-tau in Europeans, possibly related to its association with increased *MAPT* expression (Sarnowski et al., 2022). The rs242557 minor allele occurs at a relatively high frequency in European populations (ALFA frequency = 0.36), is less common in African ancestry populations (∼0.32), and is the major allele in East Asian populations (∼0.57). To our knowledge, there are no reports comparing tau pathology or tau biomarkers between European, African, and Asian ancestry individuals stratified by 17q21.31 haplotype; however, given the lack of the H2 haplotype and increased frequency of tauopathy-associated H1 variants in East Asian populations, it may be expected that there will be a differential contribution of the 17q21.31 locus to neurodegenerative risk and tau biomarkers in this population. New efforts, such as the NCRAD-ACAD study^5^ are currently underway to characterize AD biomarkers in this understudied population and will provide valuable data and insight into the validity of current AD biomarkers in Asian cohorts.

## Discussion

Genetic studies of neurodegenerative diseases have historically focused on White, European ancestry populations. However, recent efforts to expand these analyses into globally diverse populations have revealed complex ancestry and population-specific associations that may reflect unique pathogenic mechanisms underlying disease susceptibility and progression. In this review, we have focused on two loci of interest; 17q21.31 and *APOE*. Both show distinct, complex evolutionary patterns of selection that likely underlie their differential disease risk across populations. However, a thorough assessment of their contributions to AD or other dementias in non-European populations is precluded by lack of data, resulting in part from lack of infrastructure and funding, availability and accessibility of neurologists and healthcare, as well as social stigma surrounding disease (Dekker et al., 2020).

The vast majority of genetic association and biomarker analyses in non-European ancestry individuals have been carried out in admixed American populations, further complicating our understanding of genetic ancestry to neurodegenerative disease risk. African Americans and Hispanic individuals in New York City were found to have an increased risk of AD, regardless of *APOE* genotype (Maestre et al., 1995), and African Americans are consistently reported as being more susceptible to AD risk even after correcting for relevant comorbidities such as cardiovascular health and diabetes, as well as socioeconomic factors (Barnes, 2022). However, the prevalence and incidence of dementia in Africa are among the lowest in the world (Akinyemi et al., 2022). It is therefore currently unclear to what extent these disparities are due to uncharacterized lifestyle, environmental and health effects resulting from racialization in the United States, or to complex and unique genetic interactions and variants resulting from admixture. Thus, increasing representation of non-European individuals as well as integrating risk factors that reflect lived experiences and biological data will allow us to thoroughly investigate the underlying mechanisms of neurodegenerative diseases and address the existent racial differences.

Biomarkers can assist in addressing the health disparities of AD in historically disadvantaged populations via establishing generalizable diagnostics and identifying individuals in the pre-clinical stages of AD (Gleason et al., 2022). Biomarker data for AD have been inconsistent in African ancestry populations, and entirely lacking in Asian populations, largely due to small sample sizes as a result of selection biases, stigmatization, and confounding due to social determinants of physical and cognitive health. Furthermore, the rate of participation of historically disadvantaged populations in biomarker research is lower than that of European ancestry individuals in the USA, due to historical trauma of lived experiences and a continued lack of trust in the healthcare system (Gleason et al., 2022). There is, undoubtedly, work to be done, but blood-based neurodegenerative biomarkers provide ways to improve recruitment and retention of marginalized ethnic individuals, and thus, advance the diversity of neurodegenerative genomic research.

**FIGURE 3:**
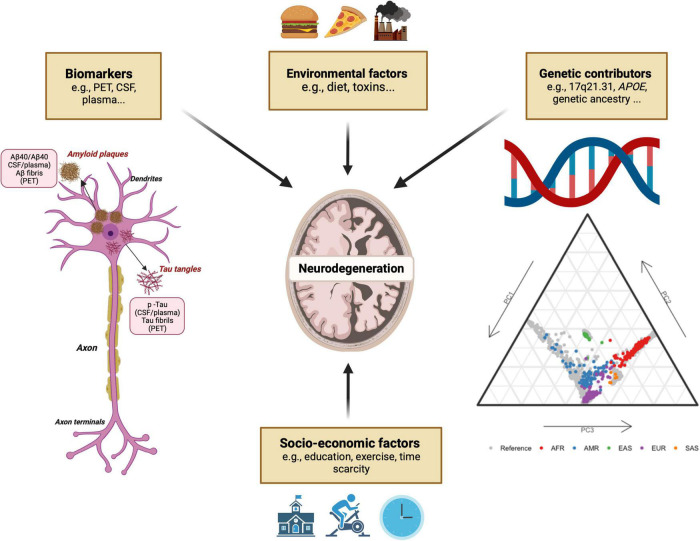
Main factors implicated in neurodegenerative disease and associated fluid biomarkers.

Race and ancestry are inextricably intertwined, particularly in USA populations, and decoding genetic contributions of ancestry across the genome will likely prove to be challenging. Given the complexity of these loci and the frequency of admixed populations, the use of racial self-identification alone or global genetic ancestry estimates to stratify populations is unlikely to provide sufficient resolution. Additional local ancestry analyses of specific risk loci, integrated with biomarker data and environmental and socio-economic factors experienced as a consequence of race will therefore be crucial to understand the complex interaction between ancestry and neurodegenerative disease risk (Figure 3). Specific programs and studies of neurodegeneration, including frequency, genetic risk, and biomarker measurements, are currently underway in non-European ancestry countries, and initiatives that pair with, and support local research facilities are becoming increasingly common. These critical efforts hold great potential to reduce racial disparities in neurodegeneration research, and subsequently incidence and prevalence.

## Conclusion

Incorporation of the amazing genetic diversity at the 17q21.31 locus and the clear differential association of *APOE* in different populations in studies of disease-associated pathways and mechanisms are required to fuel the needed interdisciplinary work necessary to promote health equity across the globe. We have discussed the complex interactions between evolutionary selection, genetic ancestry, and biomarker outcomes for two loci critical for neurodegenerative disease risk: 17q21.31 and *APOE*. Increasing representation of non-European individuals in genetic and biomarker studies will lead to an improved understanding of disease pathogenesis, and ultimately the development of therapeutic strategies that will be effective across diverse populations. We are optimistic for a future of improved inclusion of diverse populations in neurodegenerative research, such that we more accurately represent the level of diversity that exists globally. Indeed, many such important efforts are already underway.

## Author contributions

KB organized and supervised the review with the active collaboration of the rest of the authors. NH wrote about *APOE* locus. KB wrote about the 17q21.31 locus. NH and KB made the figures. All authors provided sections of text covering their area of expertise, participated in the proofreading, discussion, read, and approved the final manuscript.

## Footnotes

1
www.alzint.org

2
https://www.ebi.ac.uk/gwas/

3
https://www.ipdgc-africa.com/

4
www.alzforum.org

5
https://acadstudy.org/
