# Supplementary material for: The influence of 17q21.31 and APOE genetic ancestry on neurodegenerative disease risk
Source: Front Aging Neurosci. 2022 Oct 20;14:1021918. doi: 10.3389/fnagi.2022.1021918 (PMC9632173; doi:10.3389/fnagi.2022.1021918)
Supplement: Supplementary file 1 [file Data_Sheet_1.docx]

# **Supplementary data**

**List of references for APOE ɛ4 allele frequencies**

Abondio, P., Sazzini, M., Garagnani, P., Boattini, A., Monti, D., Franceschi, C., Luiselli, D., & Giuliani, C. (2019). The Genetic Variability of APOE in Different Human Populations and Its Implications for Longevity. *Genes (Basel)*, *10*(3). <https://doi.org/10.3390/genes10030222>

Abou Ziki, M. D., Strulovici-Barel, Y., Hackett, N. R., Rodriguez-Flores, J. L., Mezey, J. G., Salit, J., Radisch, S., Hollmann, C., Chouchane, L., Malek, J., Zirie, M. A., Jayyuosi, A., Gotto, A. M., Jr., & Crystal, R. G. (2014). Prevalence of the apolipoprotein E Arg145Cys dyslipidemia at-risk polymorphism in African-derived populations. *Am J Cardiol*, *113*(2), 302-308. <https://doi.org/10.1016/j.amjcard.2013.09.021>

Adler, G., Adler, M. A., Urbańska, A., Skonieczna-Żydecka, K., Kiseljakovic, E., Valjevac, A., Parczewski, M., & Hadzovic-Dzuvo, A. (2017). Bosnian study of APOE distribution (BOSAD): a comparison with other European populations. *Ann Hum Biol*, *44*(6), 568-573. <https://doi.org/10.1080/03014460.2017.1346708>

Al-Bustan, S. A., Alnaqeeb, M. A., Annice, B. G., Ibrhim, G., Al-Rubaian, J., Ahmed, A. H., & Refai, T. M. (2005). Apolipoprotein E genotyping among the healthy Kuwaiti population. *Hum Biol*, *77*(4), 487-498. <https://doi.org/10.1353/hub.2005.0058>

Al-Yahyaee, S. A., Al-Kindi, M. N., & Al-Bahrani, A. H. (2005). Distribution of apolipoprotein E alleles in the Omani population. *Med Princ Pract*, *14*(2), 73-78. <https://doi.org/10.1159/000083914>

Atadzhanov, M., Mwaba, M. H., Mukomena, P. N., Lakhi, S., Mwaba, P., Rayaprolu, S., Meschia, J. F., & Ross, O. A. (2014). Frequency of APOE, MTHFR and ACE polymorphisms in the Zambian population. *BMC Res Notes*, *7*, 194. <https://doi.org/10.1186/1756-0500-7-194>

Auton, A., Brooks, L. D., Durbin, R. M., Garrison, E. P., Kang, H. M., Korbel, J. O., Marchini, J. L., McCarthy, S., McVean, G. A., & Abecasis, G. R. (2015). A global reference for human genetic variation. *Nature*, *526*(7571), 68-74. <https://doi.org/10.1038/nature15393>

Bahri, R., Esteban, E., Moral, P., Hassine, M., Ben Hamda, K., & Chaabani, H. (2008). Apolipoprotein gene polymorphisms and plasma levels in healthy Tunisians and patients with coronary artery disease. *Lipids Health Dis*, *7*, 46. <https://doi.org/10.1186/1476-511x-7-46>

Bazrgar, M., Karimi, M., Fathzadeh, M., Senemar, S., Peiravian, F., Shojaee, A., & Saadat, M. (2008). Apolipoprotein E polymorphism in Southern Iran: E4 allele in the lowest reported amounts. *Mol Biol Rep*, *35*(4), 495-499. <https://doi.org/10.1007/s11033-007-9113-3>

Berkinbayev, S., Rysuly, M., Mussayev, A., Blum, K., Baitasova, N., Mussagaliyeva, A., Dzhunusbekova, G., Makhatov, B., Mussayev, A., Yeshmanova, A., Lesbekova, R., Marchuk, Y., Azhibekova, R., Oscar-Berman, M., & Kulmaganbetov, M. (2014). Apolipoprotein Gene Polymorphisms (APOB, APOC111, APOE) in the Development of Coronary Heart Disease in Ethnic Groups of Kazakhstan. *J Genet Syndr Gene Ther*, *5*(2), 216. <https://doi.org/10.4172/2157-7412.100021610.4172/2157-7412.1000216>

Borinskaia, S. A., Kal'ina, N. R., Sanina, E. D., Kozhekbaeva Zh, M., Gupalo, E., Garmash, I. V., Ogurtsov, P. P., Parshukova, O. N., Boĭko, S. G., Veselovskiĭ, E. M., Vershubskaia, G. G., Kozlov, A. I., Rogaev, E. I., & Iankovskiĭ, N. K. (2007). [Polymorphism of the apolipoprotein E gene (APOE) in the populations of Russia and neighboring countries]. *Genetika*, *43*(10), 1434-1440.

Boulenouar, H., Mediene Benchekor, S., Meroufel, D. N., Lardjam Hetraf, S. A., Ouhaibi Djellouli, H., Hermant, X., Grenier-Boley, B., Hamani Medjaoui, I., Saidi Mehtar, N., Amouyel, P., Houti, L., Meirhaeghe, A., & Goumidi, L. (2013). Impact of APOE gene polymorphisms on the lipid profile in an Algerian population. *Lipids Health Dis*, *12*, 155. <https://doi.org/10.1186/1476-511x-12-155>

Chelenkova, P., Petkova, R., Chamova, T., Zhelyazkova, S., Tournev, I., & Chakarov, S. (2018). The fine art of vascular wall maintenance. Carriership of XPC, TP53 and APOE polymorphisms may be a risk factor for cerebral vascular accidents in the Bulgarian population. *Biotechnology & Biotechnological Equipment*, *32*(6), 1558-1566. <https://doi.org/10.1080/13102818.2018.1529542>

Corbo, R. M., & Scacchi, R. (1999). Apolipoprotein E (APOE) allele distribution in the world. Is APOE*4 a 'thrifty' allele? *Ann Hum Genet*, *63*(Pt 4), 301-310. <https://doi.org/10.1046/j.1469-1809.1999.6340301.x>

Curocichin, G., Wu, Y., McDade, T. W., Kuzawa, C. W., Borja, J. B., Qin, L., Lange, E. M., Adair, L. S., Lange, L. A., & Mohlke, K. L. (2011). Single-nucleotide polymorphisms at five loci are associated with C-reactive protein levels in a cohort of Filipino young adults. *J Hum Genet*, *56*(12), 823-827. <https://doi.org/10.1038/jhg.2011.106>

Fernandes, M. A., Oliveira, C. R., Oliveira, L. M., Nogueira, A. J., Santiago, B., & Santana, I. (1999). Apolipoprotein E epsilon4 allele is a risk factor for Alzheimer's disease: the central region of portugal (Coimbra) as a case study. *Eur Neurol*, *42*(3), 183-184. <https://doi.org/10.1159/000008098>

Fernández-Mestre, M. T., Yehirobi, C., Montagnani, S., Balbas, O., & Layrisse, Z. (2005). Genetic variability of Apolipoprotein E in different populations from Venezuela. *Dis Markers*, *21*(1), 15-19. <https://doi.org/10.1155/2005/625182>

Gayà-Vidal, M., Athanasiadis, G., Carreras-Torres, R., Via, M., Esteban, E., Villena, M., Vasquez, R., Dugoujon, J. M., & Moral, P. (2012). Apolipoprotein E/C1/C4/C2 gene cluster diversity in two native Andean populations: Aymaras and Quechuas. *Ann Hum Genet*, *76*(4), 283-295. <https://doi.org/10.1111/j.1469-1809.2012.00712.x>

Kalaria, R. N., Ogeng'o, J. A., Patel, N. B., Sayi, J. G., Kitinya, J. N., Chande, H. M., Matuja, W. B., Mtui, E. P., Kimani, J. K., Premkumar, D. R., Koss, E., Gatere, S., & Friedland, R. P. (1997). Evaluation of risk factors for Alzheimer's disease in elderly east Africans. *Brain Res Bull*, *44*(5), 573-577. <https://doi.org/10.1016/s0361-9230(97)00310-9>

Khabour, O. F., & Abdelhalim, E. S. (2020). Distribution of APOE gene variations in the Jordanian population: Association with longevity. *Journal of King Saud University - Science*, *32*(1), 518-522. <https://doi.org/https://doi.org/10.1016/j.jksus.2018.08.004>

Lee, J. H., Cheng, R., Barral, S., Reitz, C., Medrano, M., Lantigua, R., Jiménez-Velazquez, I. Z., Rogaeva, E., St George-Hyslop, P. H., & Mayeux, R. (2011). Identification of novel loci for Alzheimer disease and replication of CLU, PICALM, and BIN1 in Caribbean Hispanic individuals. *Arch Neurol*, *68*(3), 320-328. <https://doi.org/10.1001/archneurol.2010.292>

Mahfouz, R. A., Sabbagh, A. S., Zahed, L. F., Mahfoud, Z. R., Kalmoni, R. F., Otrock, Z. K., Taher, A. T., & Zaatari, G. S. (2006). Apolipoprotein E gene polymorphism and allele frequencies in the Lebanese population. *Mol Biol Rep*, *33*(2), 145-149. <https://doi.org/10.1007/s11033-006-6260-x>

Marca, V., Acosta, O., Cornejo-Olivas, M., Ortega, O., Huerta, D., & Mazzetti, P. (2011). [Genetic polymorphism of apolipoprotein E in a Peruvian population]. *Rev Peru Med Exp Salud Publica*, *28*(4), 589-594. (Polimorfismo genético de la Apolipoproteína E en una población peruana.)

Marrzoq, L. F., Sharif, F. A., & Abed, A. A. (2011). Relationship between ApoE gene polymorphism and coronary heart disease in Gaza Strip. *J Cardiovasc Dis Res*, *2*(1), 29-35. <https://doi.org/10.4103/0975-3583.78584>

Masemola, M. L., Alberts, M., & Urdal, P. (2007). Apolipoprotein E genotypes and their relation to lipid levels in a rural South African population. *Scand J Public Health Suppl*, *69*, 60-65. <https://doi.org/10.1080/14034950701355635>

Midorikawa, K., Soukaloun, D., Akkhavong, K., Southivong, B., Rattanavong, O., Sengkhygnavong, V., Pyaluanglath, A., Sayasithsena, S., Nakamura, S., Midorikawa, Y., & Murata, M. (2016). APOE Genotype in the Ethnic Majority and Minority Groups of Laos and the Implications for Non-Communicable Diseases. *PLoS One*, *11*(5), e0155072. <https://doi.org/10.1371/journal.pone.0155072>

Morelli, L., Leoni, J., Castano, E. M., Mangone, C. A., & Lambierto, A. (1996). Apolipoprotein E polymorphism and late onset Alzheimer's disease in Argentina. *J Neurol Neurosurg Psychiatry*, *61*(4), 426-427. <https://doi.org/10.1136/jnnp.61.4.426>

Murrell, J. R., Price, B., Lane, K. A., Baiyewu, O., Gureje, O., Ogunniyi, A., Unverzagt, F. W., Smith-Gamble, V., Gao, S., Hendrie, H. C., & Hall, K. S. (2006). Association of apolipoprotein E genotype and Alzheimer disease in African Americans. *Arch Neurol*, *63*(3), 431-434. <https://doi.org/10.1001/archneur.63.3.431>

Petkeviciene, J., Smalinskiene, A., Luksiene, D. I., Jureniene, K., Ramazauskiene, V., Klumbiene, J., & Lesauskaite, V. (2012). Associations between apolipoprotein E genotype, diet, body mass index, and serum lipids in Lithuanian adult population. *PLoS One*, *7*(7), e41525. <https://doi.org/10.1371/journal.pone.0041525>

Rajeevan, H., Soundararajan, U., Kidd, J. R., Pakstis, A. J., & Kidd, K. K. (2012). ALFRED: an allele frequency resource for research and teaching. *Nucleic Acids Res*, *40*(Database issue), D1010-1015. <https://doi.org/10.1093/nar/gkr924>

Ramadan, A., Foda, B. M., Noha, A. S., Refaat, O., Saleh, A. A., & Fawzy, A. (2019). Association analysis of ApoE gene polymorphisms among Egyptian patients with Alzheimer's disease. *Gene Reports*, *14*, 65-71. <https://doi.org/https://doi.org/10.1016/j.genrep.2018.10.017>

Sima, A., Iordan, A., & Stancu, C. (2007). Apolipoprotein E polymorphism--a risk factor for metabolic syndrome. *Clin Chem Lab Med*, *45*(9), 1149-1153. <https://doi.org/10.1515/cclm.2007.258>

Singh, P. P., Singh, M., & Mastana, S. S. (2006). APOE distribution in world populations with new data from India and the UK. *Ann Hum Biol*, *33*(3), 279-308. <https://doi.org/10.1080/03014460600594513>

Stuart, S., Donges, B., Murrell, M., Haupt, L. M., Lea, R. A., & Griffiths, L. R. (2013). Investigation of APOE isoforms and the association between APOE E3 and E4 with migraine in the Australian Caucasian population. *Neuroreport*, *24*(10), 499-503. <https://doi.org/10.1097/WNR.0b013e3283625f55>

Tanyanyiwa, D. M., Marais, A. D., Byrnes, P., & Jones, S. (2016). The influence of ApoE genotype on the lipid profile and lipoproteins during normal pregnancy in a Southern African population. *Afr Health Sci*, *16*(3), 853-859. <https://doi.org/10.4314/ahs.v16i3.28>

They-They, T. P., Hamzi, K., Moutawafik, M. T., Bellayou, H., El Messal, M., & Nadifi, S. (2010). Prevalence of angiotensin-converting enzyme, methylenetetrahydrofolate reductase, Factor V Leiden, prothrombin and apolipoprotein E gene polymorphisms in Morocco. *Ann Hum Biol*, *37*(6), 767-777. <https://doi.org/10.3109/03014461003738850>

Willis, F., Graff-Radford, N., Pinto, M., Lawson, L., Adamson, J., Epstein, D., Parfitt, F., Hutton, M., & O'Brien, P. C. (2003). Apolipoprotein epsilon4 allele frequency in young Africans of Ugandan descent versus African Americans. *J Natl Med Assoc*, *95*(1), 71-76.

Wozniak, M. A., Faragher, E. B., Todd, J. A., Koram, K. A., Riley, E. M., & Itzhaki, R. F. (2003). Does apolipoprotein E polymorphism influence susceptibility to malaria? *J Med Genet*, *40*(5), 348-351. <https://doi.org/10.1136/jmg.40.5.348>

Zekraoui, L., Lagarde, J. P., Raisonnier, A., Gérard, N., Aouizérate, A., & Lucotte, G. (1997). High frequency of the apolipoprotein E *4 allele in African pygmies and most of the African populations in sub-Saharan Africa. *Hum Biol*, *69*(4), 575-581.

Zetterberg, M., Zetterberg, H., Palmér, M., Rymo, L., Blennow, K., Tasa, G., Juronen, E., Veromann, S., Teesalu, P., Karlsson, J. O., & Höglund, K. (2004). Apolipoprotein E polymorphism in patients with cataract. *Br J Ophthalmol*, *88*(5), 716-718. <https://doi.org/10.1136/bjo.2003.032698>
